# Supplementary material for: Dimensional evolution of charge mobility and porosity in covalent organic frameworks
Source: Nat Commun. 2025 Mar 5;16:2219. doi: 10.1038/s41467-025-57436-y (PMC11882946; doi:10.1038/s41467-025-57436-y)
Supplement: Supplementary file 1 — Supplementary Information [file 41467_2025_57436_MOESM1_ESM.pdf]

Supplementary Information for

Dimensional Evolution of Charge Mobility and Porosity in Covalent Organic Frameworks

Shuai Fu[1,#], Xiao Li[2,#], Guanzhao Wen[1], Yunyu Guo[2], Matthew A. Addicoat[3], Mischa Bonn[1], Enquan Jin[1,2*], Klaus Müllen[1*], Hai I. Wang[1,4*]

[1]Max Planck Institute for Polymer Research, Ackermannweg 10, D-55128 Mainz, Germany
[2]State Key Laboratory of Inorganic Synthesis and Preparative Chemistry, College of Chemistry and International Center of Future Science, Jilin University, Changchun 130012, P.R. China
[3]School of Science and Technology, Nottingham Trent University, Clifton Lane, Nottingham NG11 8NS, UK
[4]Nanophotonics, Debye Institute for Nanomaterials Science, Utrecht University, Princetonplein 1, 3584 CC Utrecht, The Netherlands
[#]These authors contributed equally: Shuai Fu, Xiao Li
*E-mail: enquanjin@jlu.edu.cn; muellen@mpip-mainz.mpg.de; h.wang5@uu.nl

Table of Contents

Supplementary section 1: Computational details…………………………………………………………….3
Supplementary section 2: Supplementary figures 1–15…………….……………………………………..4
Supplementary section 3: Supplementary tables 1–3…………….………………………………….....19
Supplementary references……………………………………………………………………..…..23

Supplementary section 1: Computational details

Computational details of absorption features

The simulation of absorption features was performed using DFT with the CASTEP package in Materials Studio. The Perdew-Burke-Ernzerhof (PBE) generalized gradient approximation (GGA) functional was employed to calculate the electronic exchange-correlation energies. The Grimme DFT-D correction was adopted for the van der Waals interactions due to the failure of the GGA/PBE functional to describe nonlocal dispersion forces. The cut-off energy of the plane wave basis set was set to 435 eV for geometry optimization and energy calculations.

Computational details of electronic structures

The electronic structure calculations were performed as follows: Geometries of monolayer, AA, AB, and slip-stacked 1D Pery-COF, 2D ML-Pery-COF, and 2D PL-Pery-COF were constructed using AuToGraFS[4] and 3rd order Density Functional Tight Binding (DFTB3). All atom pairs were described using standard parameters from the 3ob-3-1 parameter set including D3-BJ treatment of dispersion. Cell parameters were optimized simultaneously with the atom positions and no symmetry or constraints were applied. After optimization, it was found that some unit cells had small deviations from 90° angles, these geometries were re-optimized with fixed cell angles. A reliable symmetric K-space was employed for all calculations. Band structure and effective masses were subsequently calculated using the same calculation parameters[1]. All calculations were undertaken using AMS2021[2]. HOMO and LUMO orbitals were visualized from the optimized structures using waveplot 0.2.

Supplementary section 2: Supplementary figures 1–15

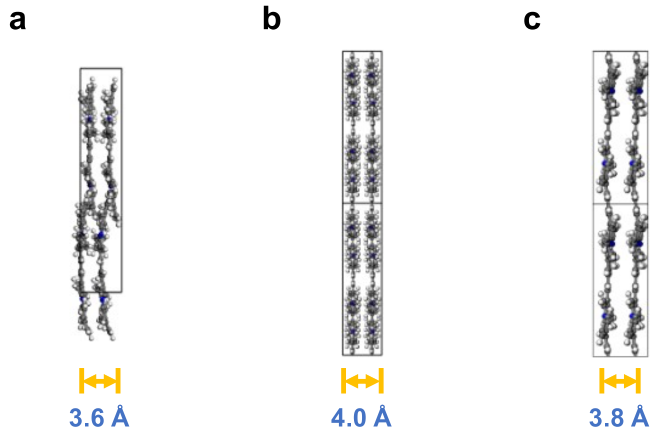

Supplementary Fig. 1 DFTB-optimized crystal structures of (**a**) 1D, (**b**) 2D PL-Pery-COF, and (**c**) 2D ML-Pery-COF viewed along the *z*-direction.

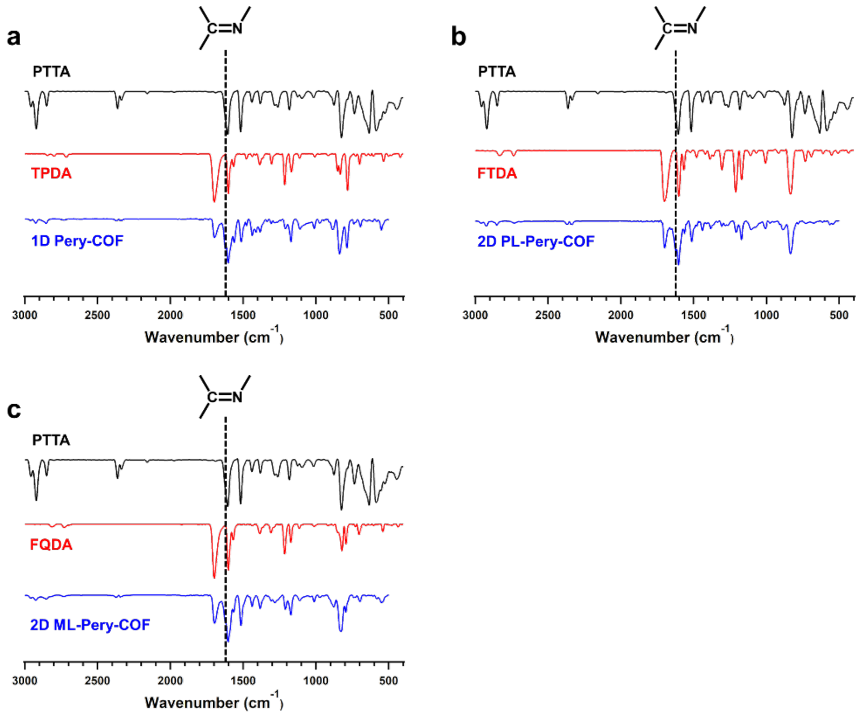

Supplementary Fig. 2 FTIR spectra of (**a**) 1D, (**b**) 2D PL-Pery-COF, and (**c**) 2D ML-Pery-COF.

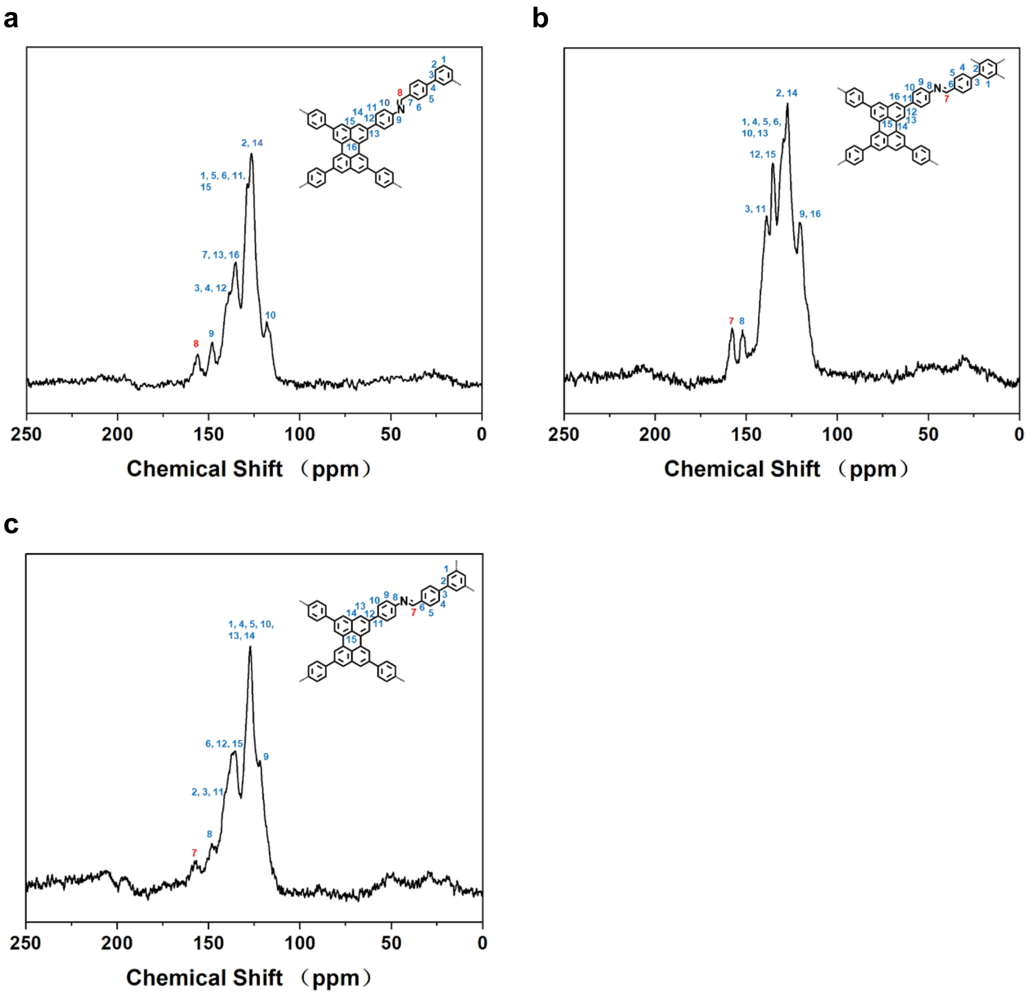

Supplementary Fig. 3 Solid-state NMR spectra of (**a**) 1D, (**b**) 2D PL-Pery-COF, and (**c**) 2D ML-Pery-COF.

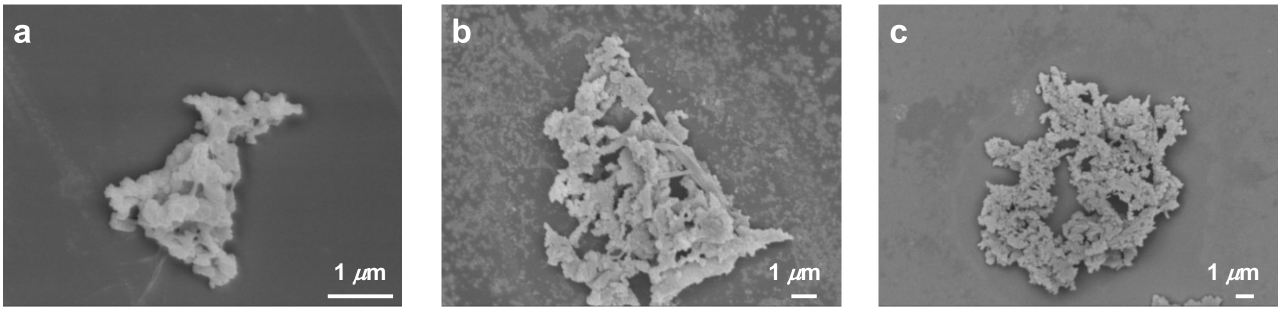

Supplementary Fig. 4 SEM images of (**a**) 1D, (**b**) 2D PL-Pery-COF, and (**c**) 2D ML-Pery-COF.

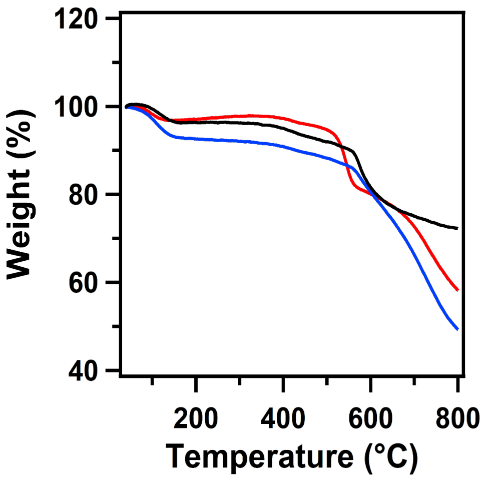

Supplementary Fig. 5 TGA of 1D (red curve), 2D PL-Pery-COF (black curve), and 2D ML-Pery-COF (blue curve).

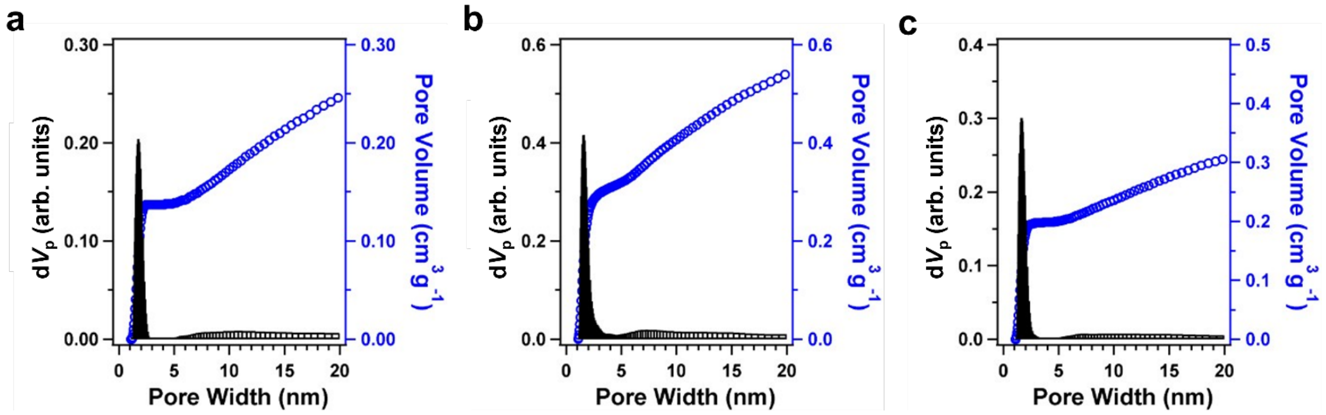

Supplementary Fig. 6 Pore size distributions of (**a**) 1D, (**b**) 2D PL-Pery-COF, and (**c**) 2D ML-Pery-COF according to QSDFT simulations.

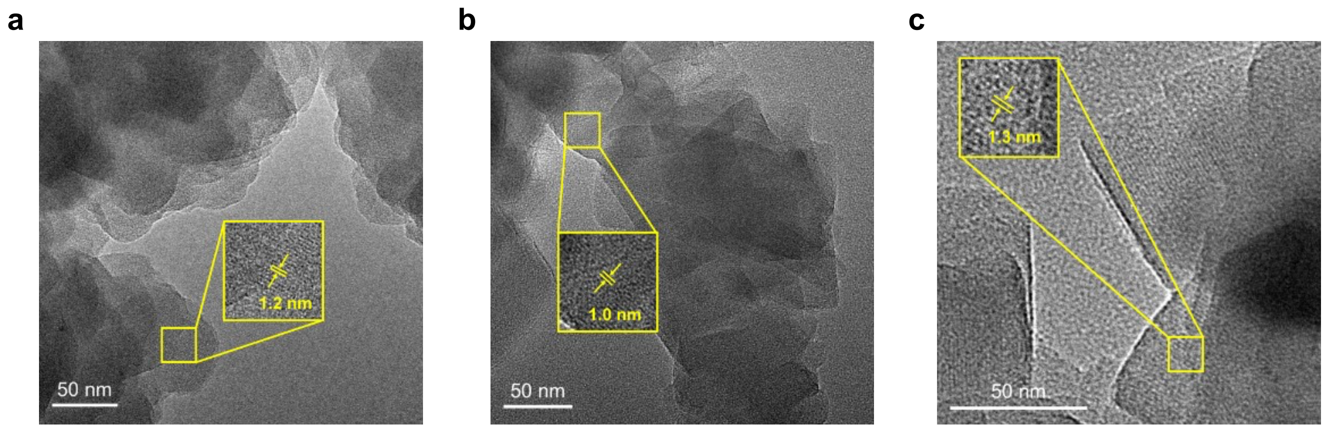

Supplementary Fig. 7 HR-TEM images of (**a**) 1D, (**b**) 2D PL-Pery-COF, and (**c**) 2D ML-Pery-COF.

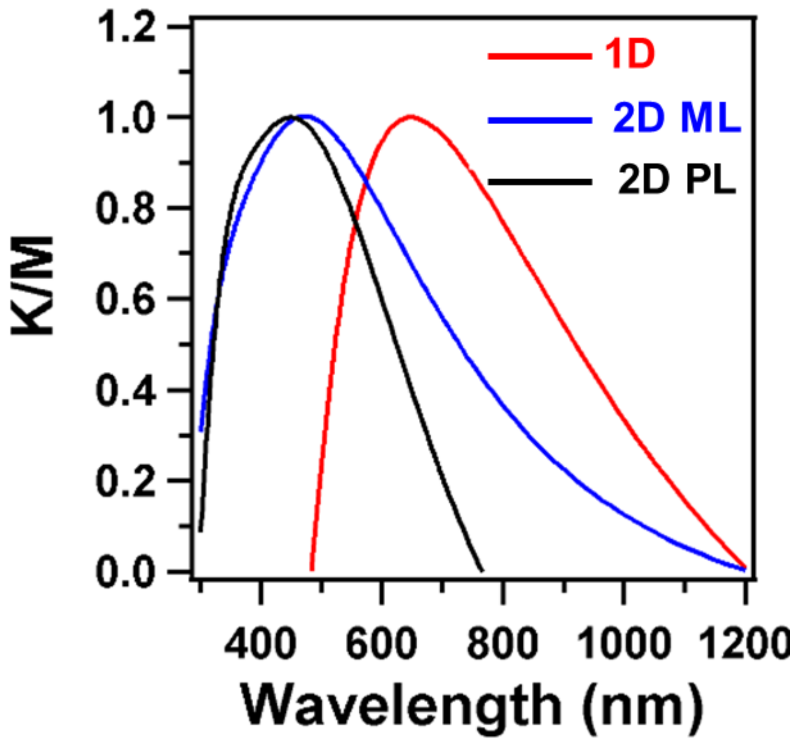

Supplementary Fig. 8 Simulated absorption spectra of 1D (red curve), 2D ML-Pery-COF (blue curve), and 2D PL-Pery-COF (black curve).

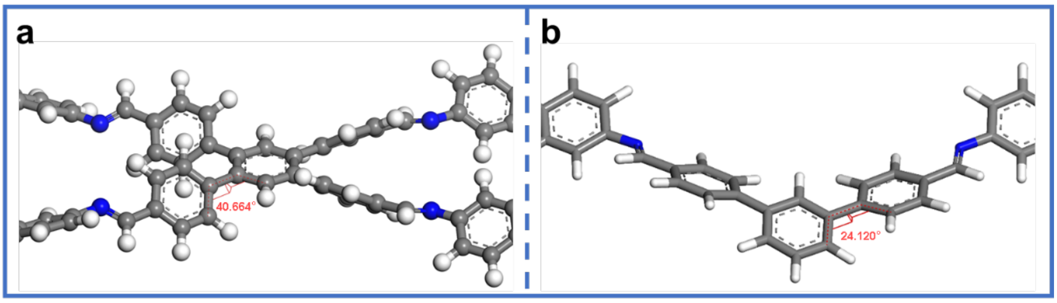

Supplementary Fig. 9 Molecular configurations of (**a**) 2D PL-Pery-COF and (**b**) 1D Pery-COF.

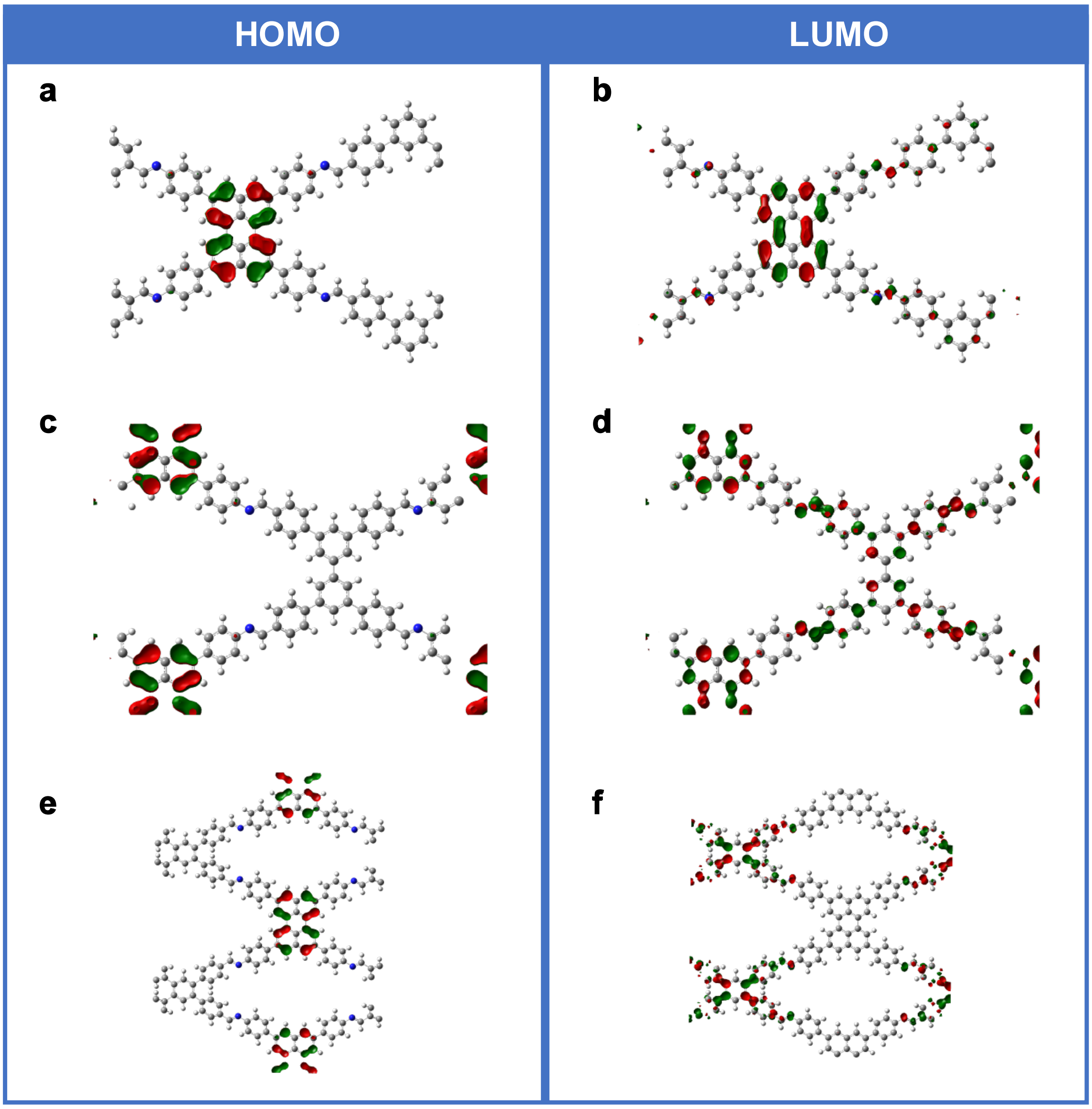

Supplementary Fig. 10 HOMO and LUMO distribution maps of (**a**–**b**) 1D, (**c**–**d**) 2D ML-Pery-COF, and (**e**–**f**) 2D PL-Pery-COF, depicted at the 0.02 isosurface.

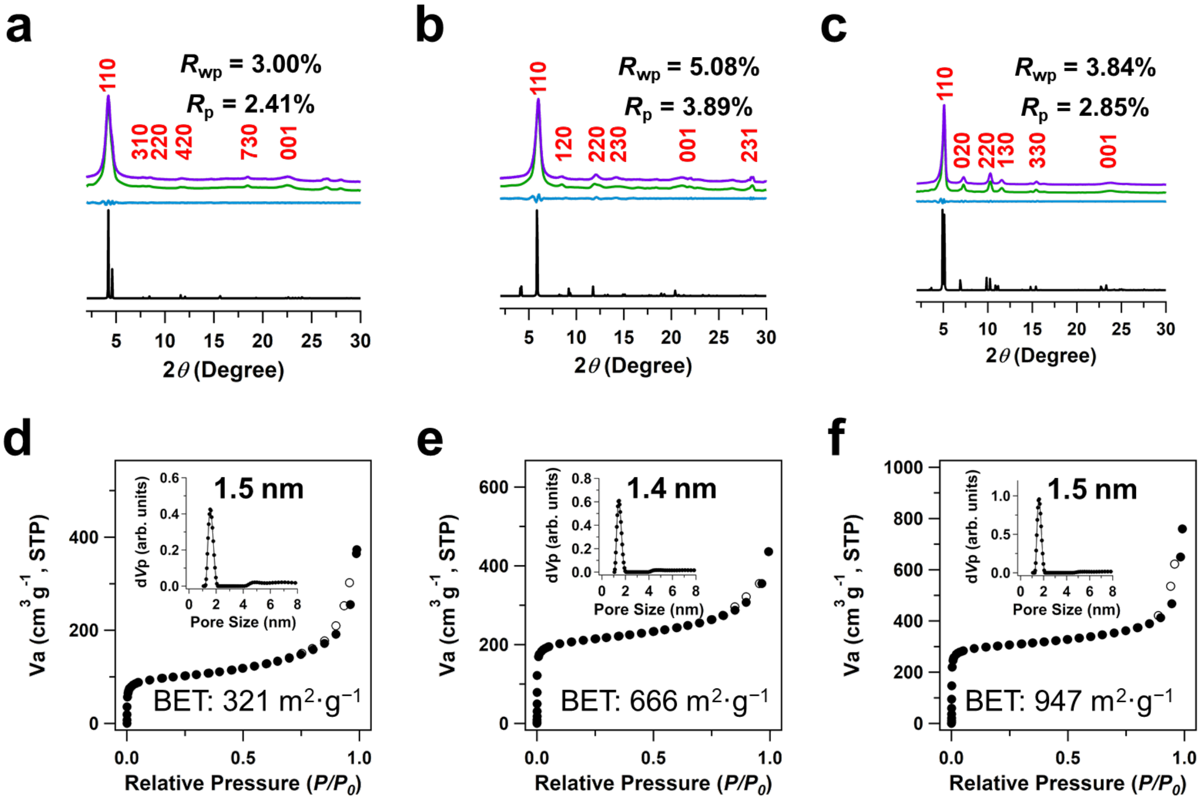

Supplementary Fig. 11 a–c Powder X-ray diffraction (PXRD) of experimentally observed (purple curves), Pawley refined (green curves), their difference (blue curves), and simulated AA stacking (black curves) patterns of (**a**) 1D Py-COF, (**b**) 2D PL-Py-COF, and (**c**) 2D ML-Py-COF; **d**–**e** Surface area and pore size distributions of (**d**) 1D Py-COF, (**e**) 2D PL-Py-COF, and (**f**) 2D ML-Py-COF.

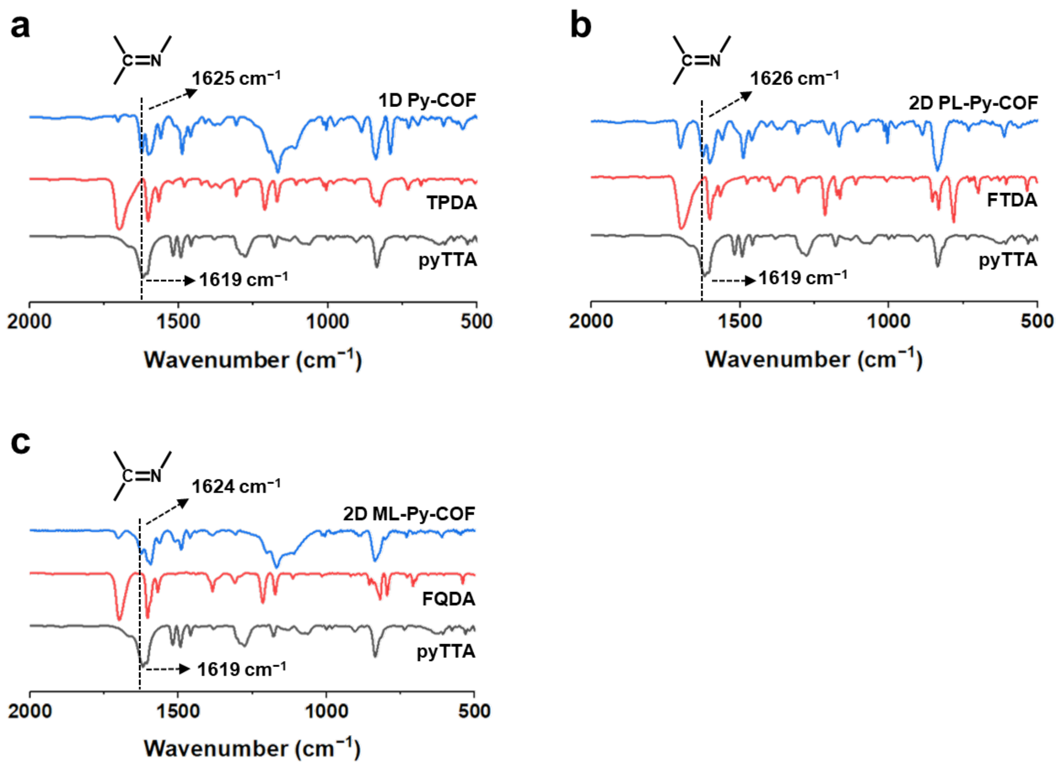

Supplementary Fig. 12 FTIR spectra of (**a**) 1D, (**b**) 2D PL-Py-COF, and (**c**) 2D ML-Py-COF.

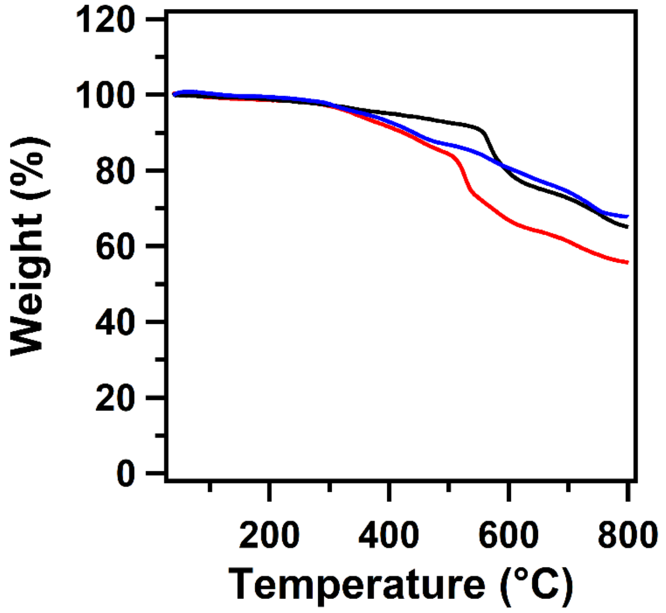

Supplementary Fig. 13 TGA of 1D Py-COF (red curve), 2D ML-Py-COF (blue curve), and 2D PL-Py-COF (black curve).

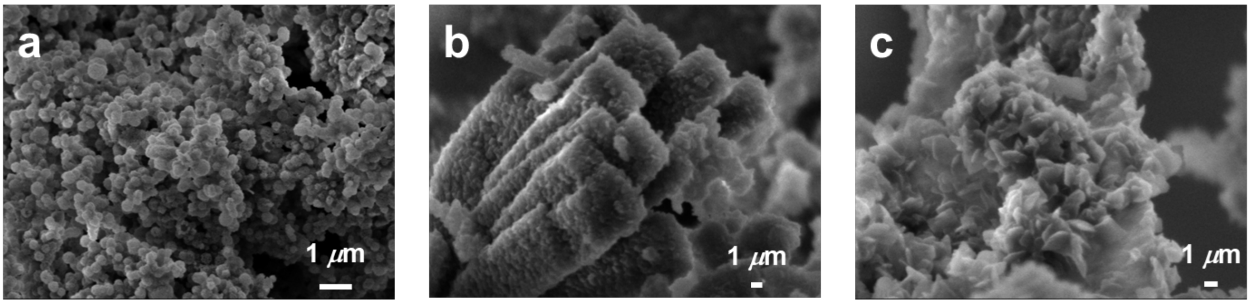

Supplementary Fig. 14 SEM images of (**a**) 1D, (**b**) 2D PL-Py-COF, and (**c**) 2D ML-Py-COF.

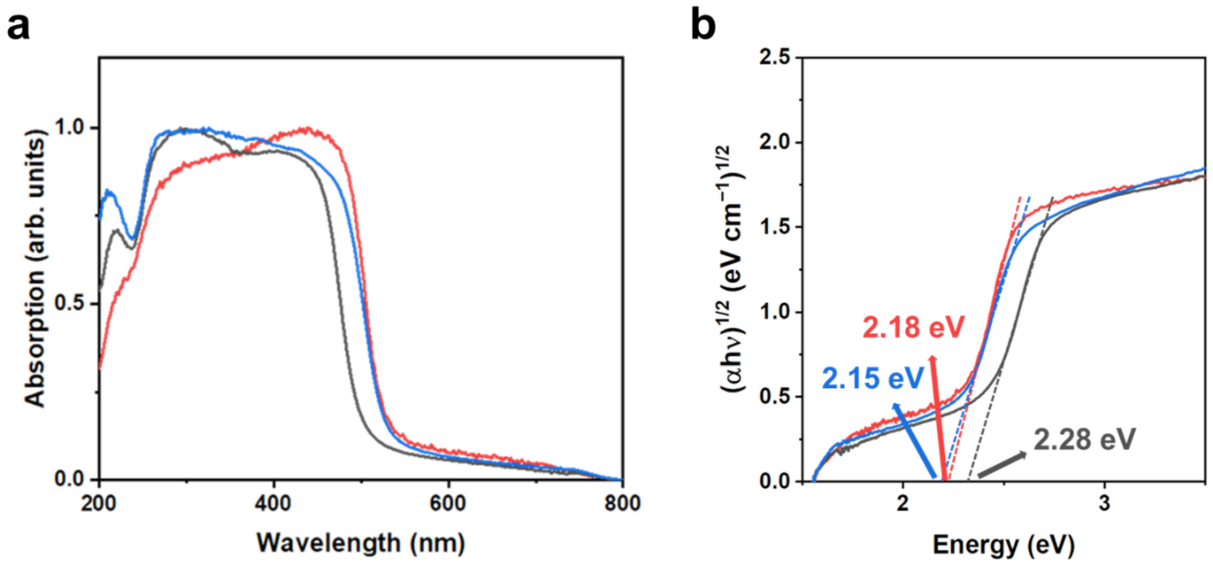

Supplementary Fig. 15 (**a**) Solid-state UV/vis diffuse reflectance spectra of 1D (red curve), 2D ML-Py-COF (blue curve), and 2D PL-Py-COF (grey curve). (**b**) Tauc plots showing the band gaps determined from the Kubelka-Munk-transformed (KM) reflectance spectra.

Supplementary section 3: Supplementary tables 1–3

Supplementary Table 1. Elemental analysis of Pery-COFs.

Sample	Value	C (%)	H (%)	N (%)
1D Pery-COF	Calculated value	90.27	4.19	5.54
	Observed value	90.44	4.69	4.87
2D ML-Pery-COF	Calculated value	90.27	4.19	5.54
	Observed value	89.68	4.63	5.65
2D PL-Pery-COF	Calculated value	90.32	4.28	5.40
	Observed value	90.00	4.57	5.43

Supplementary Table 2. DFTB calculated LUMO and HOMO energy levels (with respect to the vacuum level) and electron-hole reduced effective masses of AA stacked 1D, 2D ML-Pery-COF, and 2D PL-Pery-COF.

	**LUMO (eV)**	**HOMO (eV)**	**Electron mass (m_0)**	**Hole mass (m_0)**
**1D Pery-COF**	−3.01	−4.42	In-plane<br>2.867<br>Out-of-plane<br>1.929	2.211
**2D ML-Pery-COF**	−3.23	−4.95	In-plane<br>4.066<br>Out-of-plane<br>1.212	5.182
**2D PL-Pery-COF**	−3.37	−5.04	In-plane<br>2.752<br>Out-of-plane<br>7.427	16.237

Note that the hole masses presented here represent in-plane hole masses. Out-of-plane hole masses are not considered due to their significantly larger values, which indicate unfavorable out-of-plane hole transport.

Supplementary Table 3. A comparison of surface areas and charge mobilities of conductive COFs.

Sample	τ (fs)	μ ($cm^2 \cdot V^{-1} \cdot s^{-1}$)	A ($m^2 \cdot g^{-1}$)	Ref
1D-Pery-COF	43 ± 4	66 ± 14[a]	370	This work
2D ML- Pery-COF	43 ± 4	49 ± 10[a]	947	This work
2D PL-Pery-COF	43 ± 4	21 ± 4[a]	944	This work
1D Py-COF	N/A	N/A	321	This work
2D ML-Py-COF	N/A	N/A	947	This work
2D PL-Py-COF	N/A	N/A	666	This work
CuPc-MIDA-COF	23 ± 13	13.3 ± 7.5[a]	442	3
CuPc-MIDA-COF	N/A	8.3[b]	442	3
HHTP-MIDA-COF	16 ± 12	3.4 ± 2.5[a]	1170	3
H_2P-COF	N/A	3.5[c]	1894	4
CuP-COF	N/A	0.19[c]	1713	4
ZnP-COF	N/A	0.048[c]	1724	4
NiPc-COF	N/A	1.3[c]	624	5
NiPc-BTDA COF	N/A	0.6[c]	877	6
COF-366	N/A	8.1[c]	735	7
COF-66	N/A	3[c]	360	7
2D D-A COF	N/A	0.05[c]	2021	8
CS-COF	N/A	4.2[c]	776	9
TTF-Ph-COF	N/A	0.2[c]	1014	10
TTF-Py-COF	N/A	0.08[c]	817	10
HBC-COF (film)	N/A	0.7[c]	965	11
CuPc-pz COF	N/A	0.9 ± 0.2[b]	458.9	12
ZnPc-pz COF	N/A	4.8 ± 0.7[b]	487.4	12
NiPc-CoTAA (film)	N/A	0.15[b]	186	13
BUCT-COF-1	N/A	2.75 ± 0.22[b]	976.6	14
DBOV-COF (film)	36 ± 6	0.6 ± 0.1[d]	581	15
sp^2c-COF	41 ± 5	22.1 ± 2.7[d]	N/A	16
sp^2c-COF-6	N/A	2.3 ± 0.5[d]	667	17
sp^2c-COF-8	N/A	< 0.1[d]	569	17
sp^2c-COF-9	N/A	5.8 ± 0.9[d]	755	17
DHP-COF	28 ± 6	N/A	2031	18
c-HBC-COF	87 ± 5	44[d]	1098	18
TPB-TFB COF (film)	72	165 ± 10[d]	1232	19

V-2D-COF-W1	49 ± 8	1.4[d]	245	20
V-2D-COF-W3	37 ± 6	10.3[d]	118	20
V-2D-COF-W4	56 ± 8	0.6[d]	333	20
2DPAV-BDT-BT	33 ± 4	65[d]	N/A	21
2DPAV-BDT-BP	36 ±5	17[d]	N/A	21
2DCP-NiPc (film)	76 ±3	971 ± 44[d]	138	22
2DCP-CuPc (film)	45 ±3	460 ± 31[d]	191	22
AntTTH	N/A	~0.1	940	23

[a]Mobility obtained by time-resolved THz spectroscopy without taking into account the backscattering effect; [b]Mobility obtained by Hall effect measurements; [c]Mobility obtained by flash-photolysis time-resolved microwave conductivity; [d]Mobility obtained by time-resolved THz spectroscopy taking into account the backscattering effect.

Supplementary references

1. Michael, G., Albrecht, G. & Marcus E. Parametrization and benchmark of DFTB3 for organic molecules. *J. Chem. Theory Comput.*, **9**, 338–354 (2013).
2. AMS 2022.1, SCM, Theoretical Chemistry, Vrije Universiteit, Amsterdam, The Netherlands, http://www.scm.com.
3. Enquan, J. *et al*. Exceptional electron conduction in two-dimensional covalent organic frameworks. *Chem* **7**, 3309–3324 (2021).
4. Xiao, F. *et al.* High-rate charge-carrier transport in porphyrin covalent organic frameworks: switching from hole to electron to ambipolar conduction. *Angew. Chem. Int. Ed.* **51**, 2618–2622 (2012).
5. Chumakov, Y. *et al.* First-principles study of thermoelectric properties of covalent organic frameworks. *J. Electron. Mater.* **45**, 3445–3452 (2016).
6. Xuesong, D. *et al*. An n-channel two-dimensional covalent organic framework. *J. Am. Chem. Soc.* **133**, 14510–14513 (2011).
7. Shun, W. *et al*. Covalent organic frameworks with high charge carrier mobility. *Chem. Mater.* **23**, 4094–4097 (2011).
8. Xiao, F. *et al*. An ambipolar conducting covalent organic framework with self-sorted and periodic electron donor-acceptor ordering. *Adv. Mater.* **24**, 3026–3031 (2012).
9. Guo, J. *et al*. Conjugated organic framework with three-dimensionally ordered stable structure and delocalized π clouds. *Nat. Commun.* **4**, 2736 (2013).
10. Shangbin, J. *et al.* Two-dimensional tetrathiafulvalene covalent organic frameworks: towards latticed conductive organic salts. *Chem. Eur. J.* **20**, 14608–14613 (2014).
11. Dalapati, S. *et al.* Rational design of crystalline supermicroporous covalent organic frameworks with triangular topologies. *Nat. Commun.* **6**, 7786 (2015).
12. Chongqing, Y. *et al.* Chemically stable polyarylether-based metallophthalocyanine frameworks with high carrier mobilities for capacitive energy storage. *J. Am. Chem. Soc.* **143**, 17701–17707 (2021).
13. Yan, Y. *et al.* Conductive metallophthalocyanine framework films with high carrier mobility as efficient chemiresistors. *Angew. Chem. Int. Ed.* **60**, 10806–10813 (2021).
14. Shitao, W. *et al.* A fully conjugated 3D covalent organic framework exhibiting band-like transport with ultrahigh electron mobility. *Angew. Chem. Int. Ed.* **60**, 9321–9325 (2021).
15. Enquan, J. *et al.* A nanographene-based two-dimensional covalent organic framework as a stable and efficient photocatalyst. *Angew. Chem. Int. Ed.* **61**, e202114059 (2022).
16. Enquan, J. *et al.* Two-dimensional sp^2 carbon-conjugated covalent organic frameworks. *Science* **357**, 673–676 (2017).
17. Enquan J. *et al.* Module-patterned polymerization towards crystalline 2D sp^2-carbon covalent organic framework semiconductors. *Angew. Chem. Int. Ed.* **61**, e202115020 (2022).
18. Guolong, X, *et al.* Nonplanar rhombus and kagome 2D covalent organic frameworks from distorted aromatics for electrical conduction. *J. Am. Chem. Soc.* **144**, 5042–5050 (2022).
19. Shuai, F. *et al.* Outstanding charge mobility by band transport in two-dimensional semiconducting covalent organic frameworks. *J. Am. Chem. Soc.* **144**, 7489–7496 (2022).
20. Yannan, L. *et al.* Vinylene-linked 2D conjugated covalent organic frameworks by Wittig reactions. *Angew. Chem. Int. Ed.* **61**, e202209762 (2022).
21. Yamei, L. *et al.* A thiophene backbone enables two-dimensional poly(arylenevinylene)s with high charge carrier mobility. *Angew. Chem. Int. Ed.* **62**, e202305978 (2023).
22. Wang, M. *et al.* Exceptionally high charge mobility in phthalocyanine-based poly(benzimidazobenzophenanthroline)-ladder-type two-dimensional conjugated polymers. *Nat. Mater.* **22**, 880–887 (2023).
23. Samrat, G. *et al.* Low band gap semiconducting covalent organic framework films with enhanced photocatalytic hydrogen evolution. *J. Mater. Chem. A*, **12**, 247–255 (2024).
